# The genome sequence of an ichneumonid wasp, *Ophion costatus *Ratzeburg, 1848

**DOI:** 10.12688/wellcomeopenres.21216.1

**Published:** 2024-04-12

**Authors:** Gavin R. Broad, Chris Fletcher, Inez Januszczak, Natural History Museum Genome Acquisition Lab, Darwin Tree of Life Barcoding collective, Wellcome Sanger Institute Tree of Life Management, Samples and Laboratory team, Wellcome Sanger Institute Scientific Operations: Sequencing Operations, Wellcome Sanger Institute Tree of Life Core Informatics team, Tree of Life Core Informatics collective, Darwin Tree of Life Consortium

**Affiliations:** 1 Natural History Museum, London, England, UK

**Keywords:** Ophion costatus, ichneumonid wasp, genome sequence, chromosomal, Hymenoptera

## Abstract

We present a genome assembly from an individual male *Ophion costatus* (ichneumonid wasp; Arthropoda; Insecta; Hymenoptera; Ichneumonidae). The genome sequence is 519.4 megabases in span. Most of the assembly is scaffolded into 13 chromosomal pseudomolecules. The mitochondrial genome has also been assembled and is 25.95 kilobases in length.

## Species taxonomy

Eukaryota; Opisthokonta; Metazoa; Eumetazoa; Bilateria; Protostomia; Ecdysozoa; Panarthropoda; Arthropoda; Mandibulata; Pancrustacea; Hexapoda; Insecta; Dicondylia; Pterygota; Neoptera; Endopterygota; Hymenoptera; Apocrita; Ichneumonoidea; Ichneumonidae; Ophioninae; Ophion group; *Ophion*; *Ophion costatus* Ratzeburg, 1848 (NCBI:txid495356).

## Background

*Ophion costatus* is a relatively large (wing length up to 19 mm) nocturnal ichneumonid wasp, or Darwin Wasp. As with the great majority of *Ophion* species, *O. costatus* is predominantly testaceous, a pale reddish-yellow colour, with long antennae and large eyes, adaptations for its nocturnal behaviour. The general appearance is very similar to many other *Ophion*, and there are 32 species now known from Britain, a result of the excellent taxonomic work of Brock (1982) and then Johansson and Cederberg (2019). Despite the delimitation of increasingly well-defined *Ophion* species, identification is often difficult. *Ophion costatus* keys to *O. parvulus* in Brock (1982), but Johansson and Cederberg (2019) disentangled five species which had been confused under the name ‘ *O. parvulus*’, with *O. costatus* being one of them. The *parvulus* species group, which seems to be monophyletic ( Schwarzfeld *et al.*, 2016), generally have a rather stout first metasomal segment, a gently arched fore wing vein *RS* and the propodeum with well developed, curved transverse carinae and weak longitudinal carinae. Within this complex, *O. costatus* can be distinguished by its rather thicker tarsi, large size, pattern of the propodeal carinae and, often, by some brown patches on the body ( Johansson & Cederberg, 2019).

As with most other *Ophion* species, noctuid moths are the hosts, searched for at night, with oviposition into a late instar larva and the host consumed once it has prepared its pupation retreat ( Broad *et al.*, 2018). Little is known of the ecology of *O. costatus* but it seems to be widespread in deciduous woodland in Britain, at least in the South, and easily light-trapped from August to October. Hosts are hairy larvae of *Acronicta* species, judging by the few published rearings, from *Acronicta aceris* (the Sycamore) and *A. leporina* (the Miller) ( Brock, 1982; Johansson & Cederberg, 2019).

Rather confusingly, the name *Ophion costatus* had, until 2019, been used for different species of *Ophion*; Brock (1982) used the name ‘ *O. costatus*’ for a species-pair which were described as *Ophion brocki* and *O. splendens* by Johansson and Cederberg (2019). The availability of genomes for multiple closely related species will help us to understand the mechanisms behind host shifts in a genus notable for having many morphologically very similar species but each with tightly defined, narrow host ranges ( Shaw & Broad, 2023).

## Genome sequence report

**Figure 1.**
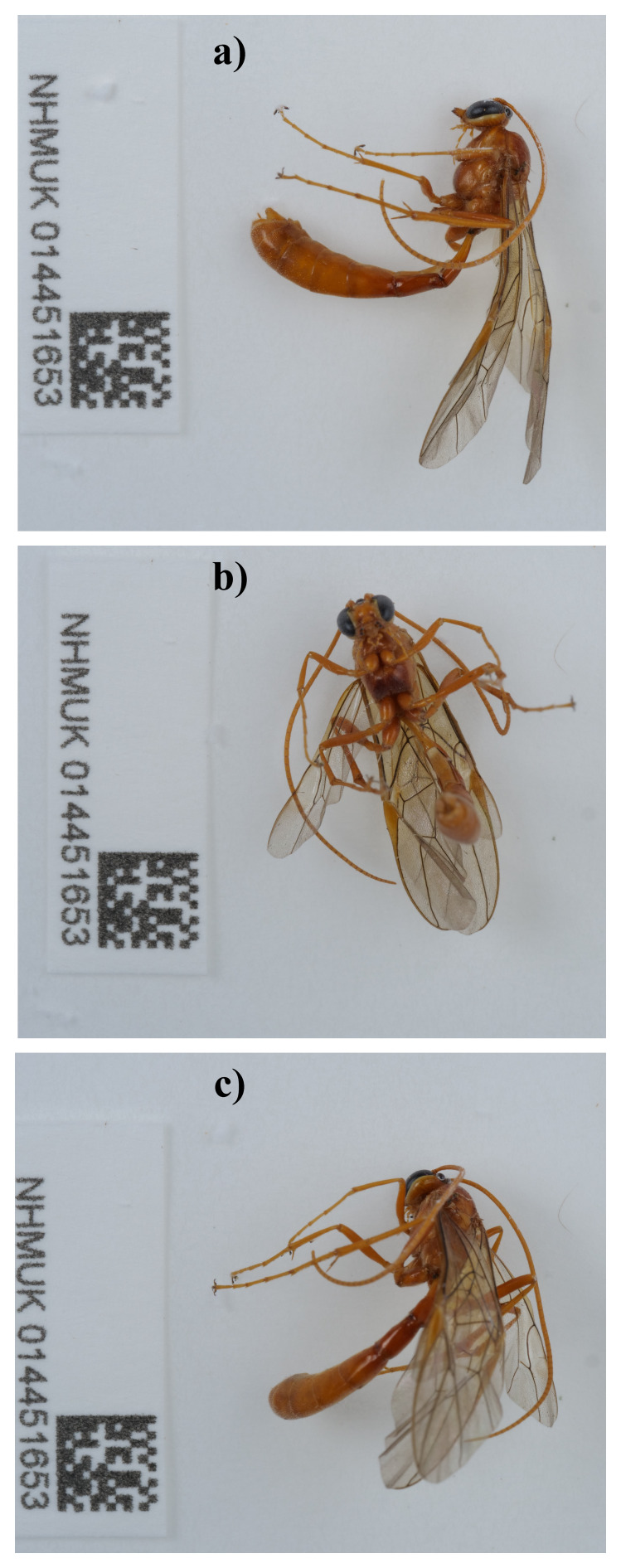
Photograph of the *Ophion costatus* (iyOphCost1) specimen used for genome sequencing.

The genome was sequenced from one male *Ophion costatus* ( Figure 1) collected from Wytham Woods, Oxfordshire, UK (51.77, –1.31). A total of 51-fold coverage in Pacific Biosciences single-molecule HiFi long reads was generated. Primary assembly contigs were scaffolded with chromosome conformation Hi-C data. Manual assembly curation corrected 172 missing joins or mis-joins, reducing the scaffold number by 26.97%, and increasing the scaffold N50 by 13.96%.

**Figure 2.**
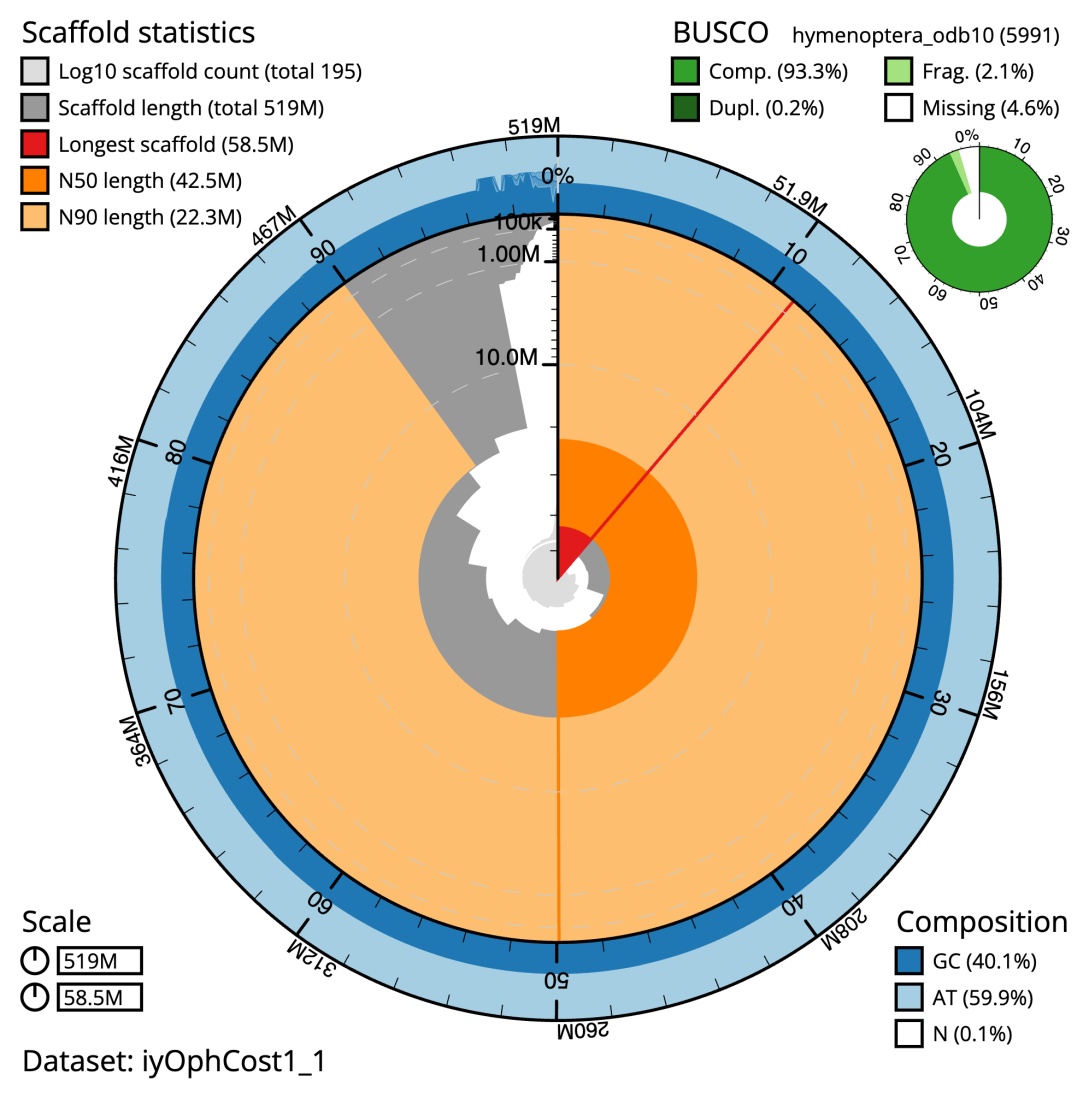
Genome assembly of *Ophion costatus*, iyOphCost1.1: metrics. The BlobToolKit Snailplot shows N50 metrics and BUSCO gene completeness. The main plot is divided into 1,000 size-ordered bins around the circumference with each bin representing 0.1% of the 519,402,440 bp assembly. The distribution of scaffold lengths is shown in dark grey with the plot radius scaled to the longest scaffold present in the assembly (58,508,097 bp, shown in red). Orange and pale-orange arcs show the N50 and N90 scaffold lengths (42,526,767 and 22,300,145 bp), respectively. The pale grey spiral shows the cumulative scaffold count on a log scale with white scale lines showing successive orders of magnitude. The blue and pale-blue area around the outside of the plot shows the distribution of GC, AT and N percentages in the same bins as the inner plot. A summary of complete, fragmented, duplicated and missing BUSCO genes in the hymenoptera_odb10 set is shown in the top right. An interactive version of this figure is available at https://blobtoolkit.genomehubs.org/view/iyOphCost1_1/dataset/iyOphCost1_1/snail.

**Figure 3.**
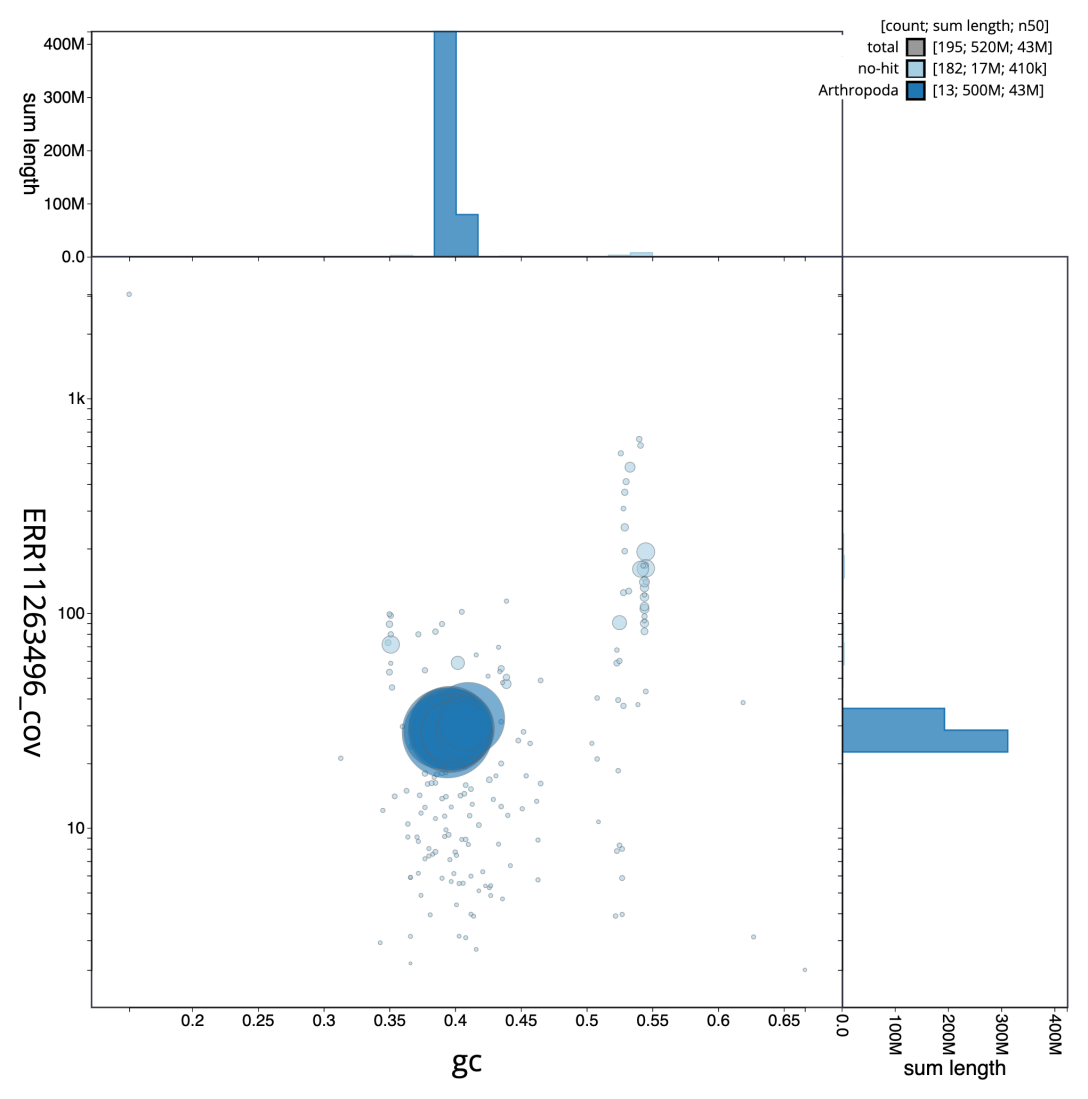
Genome assembly of *Ophion costatus*, iyOphCost1.1: BlobToolKit GC-coverage plot. Sequences are coloured by phylum. Circles are sized in proportion to sequence length. Histograms show the distribution of sequence length sum along each axis. An interactive version of this figure is available at https://blobtoolkit.genomehubs.org/view/iyOphCost1_1/dataset/iyOphCost1_1/blob.

**Figure 4.**
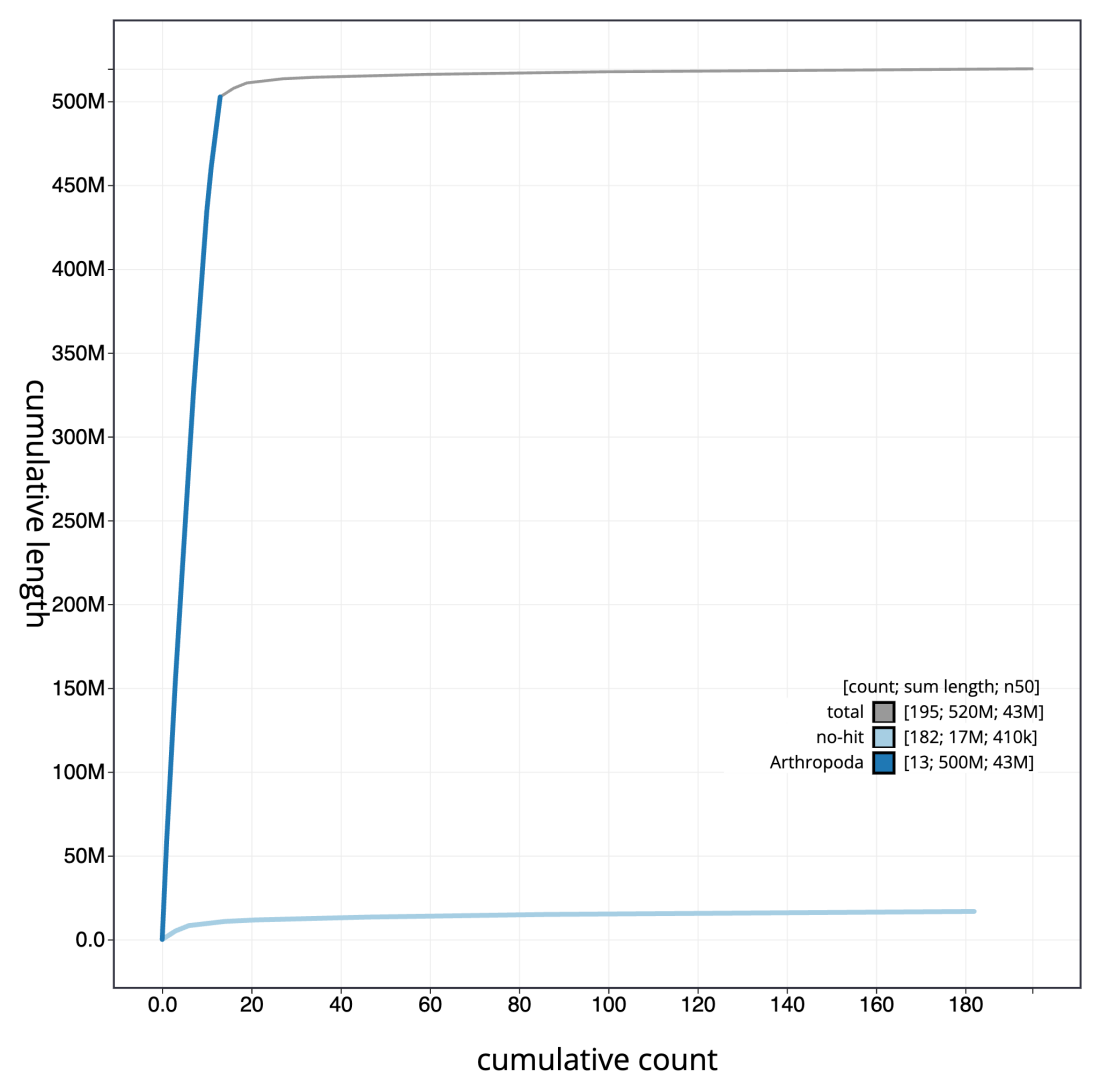
Genome assembly of *Ophion costatus*, iyOphCost1.1: BlobToolKit cumulative sequence plot. The grey line shows cumulative length for all sequences. Coloured lines show cumulative lengths of sequences assigned to each phylum using the buscogenes taxrule. An interactive version of this figure is available at https://blobtoolkit.genomehubs.org/view/iyOphCost1_1/dataset/iyOphCost1_1/cumulative.

**Figure 5.**
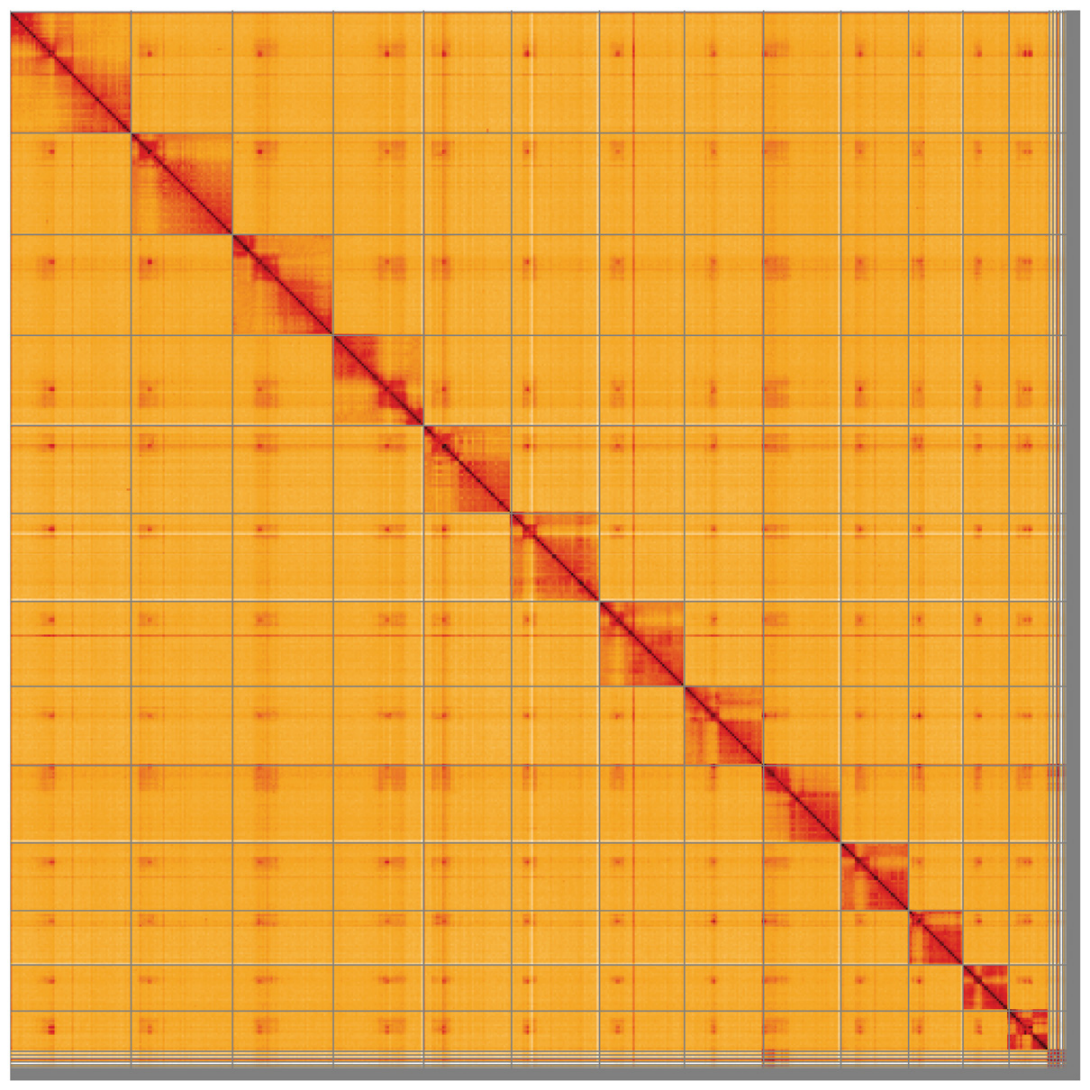
Genome assembly of *Ophion costatus*, iyOphCost1.1: Hi-C contact map of the iyOphCost1.1 assembly, visualised using HiGlass. Chromosomes are shown in order of size from left to right and top to bottom. An interactive version of this figure may be viewed at https://genome-note-higlass.tol.sanger.ac.uk/l/?d=UJ3G6fGuSDSNy81KN6pBdg.

The final assembly has a total length of 519.4 Mb in 194 sequence scaffolds with a scaffold N50 of 42.5 Mb ( Table 1). The snail plot in Figure 2 provides a summary of the assembly statistics, while the distribution of assembly scaffolds on GC proportion and coverage is shown in Figure 3. The cumulative assembly plot in Figure 4 shows curves for subsets of scaffolds assigned to different phyla. Most (99.31%) of the assembly sequence was assigned to 13 chromosomal-level scaffolds. The assembly is of a haploid male wasp. Chromosome-scale scaffolds confirmed by the Hi-C data are named in order of size ( Figure 5; Table 2). The exact order and orientation of the repetitive region on the N-terminus of chromosome 2 is unknown and its fragments have been left as unlocalised to chromosome 2. The *Ophion slaviceki* (GCA_944452715.1) ( Broad *et al.*, 2023) assembly was used to guide curation. The mitochondrial genome was also assembled and can be found as a contig within the multifasta file of the genome submission.

**Table 1.** Genome data for *Ophion costatus*, iyOphCost1.1.

Project accession data		
Assembly identifier	iyOphCost1.1	
Species	*Ophion costatus*	
Specimen	iyOphCost1	
NCBI taxonomy ID	495356	
BioProject	PRJEB61491	
BioSample ID	SAMEA111458581	
Isolate information	iyOphCost1, male: head and thorax (DNA and Hi-C sequencing); abdomen (RNA sequencing)	
Assembly metrics *		*Benchmark*
Consensus quality (QV)	56.9	*≥ 50*
*k*-mer completeness	99.99%	*≥ 95%*
BUSCO **	C:93.3%[S:93.1%,D:0.2%], F:2.1%,M:4.6%,n:5,991	*C ≥ 95%*
Percentage of assembly mapped to chromosomes	99.31%	*≥ 95%*
Sex chromosomes	None	*localised homologous * *pairs*
Organelles	Mitochondrial genome: 25.95 kb	*complete single alleles*
Raw data accessions		
PacificBiosciences SEQUEL II	ERR11263496	
Hi-C Illumina	ERR11271515	
PolyA RNA-Seq Illumina	ERR12245562	
Genome assembly		
Assembly accession	GCA_951751655.1	
Span (Mb)	519.4	
Number of contigs	1,855	
Contig N50 length (Mb)	0.6	
Number of scaffolds	194	
Scaffold N50 length (Mb)	42.5	
Longest scaffold (Mb)	58.51	

* Assembly metric benchmarks are adapted from column VGP-2020 of “Table 1: Proposed standards and metrics for defining genome assembly quality” from Rhie *et al.* (2021). ** BUSCO scores based on the hymenoptera_odb10 BUSCO set using version 5.3.2. C = complete [S = single copy, D = duplicated], F = fragmented, M = missing, n = number of orthologues in comparison. A full set of BUSCO scores is available at https://blobtoolkit.genomehubs.org/view/iyOphCost1_1/dataset/iyOphCost1_1/busco.

**Table 2.** Chromosomal pseudomolecules in the genome assembly of *Ophion costatus*, iyOphCost1.

INSDC accession	Chromosome	Length (Mb)	GC%
OX632356.1	1	58.51	39.5
OX632357.1	2	37.43	41.0
OX632358.1	3	49.06	39.5
OX632359.1	4	48.77	39.5
OX632360.1	5	43.65	40.0
OX632361.1	6	42.54	39.5
OX632362.1	7	42.53	39.5
OX632363.1	8	41.08	39.5
OX632364.1	9	38.28	39.0
OX632365.1	10	32.76	40.0
OX632366.1	11	26.26	39.5
OX632367.1	12	22.3	40.5
OX632368.1	13	19.49	41.0
OX632369.1	MT	0.03	15.0

The estimated Quality Value (QV) of the final assembly is 56.9 with *k*-mer completeness of 99.99%, and the assembly has a BUSCO v5.3.2 completeness of 93.3% (single = 93.1%, duplicated = 0.2%), using the hymenoptera_odb10 reference set ( *n* = 5,991).

Metadata for specimens, barcode results, spectra estimates, sequencing runs, contaminants and pre-curation assembly statistics are given at https://links.tol.sanger.ac.uk/species/495356.

## Methods

### Sample acquisition and nucleic acid extraction

A male *Ophion costatus* (specimen ID NHMUK014451653, ToLID iyOphCost1) was collected from Wytham Woods, Oxfordshire (biological vice-county Berkshire), UK (latitude 51.77, longitude –1.31) on 2021-09-02 using an aerial net. The specimen was collected by Gavin Broad, Chris Fletcher and Inez Januszczak (all from the Natural History Museum), identified by Gavin Broad, and then preserved by dry freezing at –80 °C.

The workflow for high molecular weight (HMW) DNA extraction at the Wellcome Sanger Institute (WSI) includes a sequence of core procedures: sample preparation; sample homogenisation, DNA extraction, fragmentation, and clean-up. In sample preparation, the iyOphCost1 sample was weighed and dissected on dry ice ( Jay *et al.*, 2023). Tissue from the head and thorax was homogenised using a PowerMasher II tissue disruptor ( Denton *et al.*, 2023a). HMW DNA was extracted in the WSI Scientific Operations core using the Automated MagAttract v2 protocol ( Oatley *et al.*, 2023). The DNA was sheared into an average fragment size of 12–20 kb in a Megaruptor 3 system with speed setting 31 ( Bates *et al.*, 2023). Sheared DNA was purified by solid-phase reversible immobilisation ( Strickland *et al.*, 2023): in brief, the method employs a 1.8X ratio of AMPure PB beads to sample to eliminate shorter fragments and concentrate the DNA. The concentration of the sheared and purified DNA was assessed using a Nanodrop spectrophotometer and Qubit Fluorometer and Qubit dsDNA High Sensitivity Assay kit. Fragment size distribution was evaluated by running the sample on the FemtoPulse system.

RNA was extracted from tissue from the abdomen of iyOphCost1 in the Tree of Life Laboratory at the WSI using the RNA Extraction: Automated MagMax™ *mir*Vana protocol ( do Amaral *et al.*, 2023). The RNA concentration was assessed using a Nanodrop spectrophotometer and a Qubit Fluorometer using the Qubit RNA Broad-Range Assay kit. Analysis of the integrity of the RNA was done using the Agilent RNA 6000 Pico Kit and Eukaryotic Total RNA assay.

Protocols developed by the WSI Tree of Life laboratory are publicly available on protocols.io ( Denton *et al.*, 2023b).

### Sequencing

Pacific Biosciences HiFi circular consensus DNA sequencing libraries were constructed according to the manufacturers’ instructions. Poly(A) RNA-Seq libraries were constructed using the NEB Ultra II RNA Library Prep kit. DNA and RNA sequencing was performed by the Scientific Operations core at the WSI on Pacific Biosciences SEQUEL II (HiFi) and Illumina NovaSeq 6000 (RNA-Seq) instruments. Hi-C data were also generated from remaining head and thorax tissue of iyOphCost1 using the Arima2 kit and sequenced on the Illumina NovaSeq 6000 instrument.

### Genome assembly, curation and evaluation

Assembly was carried out with Hifiasm ( Cheng *et al.*, 2021). The assembly was scaffolded with Hi-C data ( Rao *et al.*, 2014) using YaHS ( Zhou *et al.*, 2023). The assembly was checked for contamination and corrected using the gEVAL system ( Chow *et al.*, 2016) as described previously ( Howe *et al.*, 2021). Manual curation was performed using gEVAL, HiGlass ( Kerpedjiev *et al.*, 2018) and Pretext ( Harry, 2022). The mitochondrial genome was assembled using MitoHiFi ( Uliano-Silva *et al.*, 2023) and OATK ( Zhou, 2023).

A Hi-C map for the final assembly was produced using bwa-mem2 ( Vasimuddin *et al.*, 2019) in the Cooler file format ( Abdennur & Mirny, 2020). To assess the assembly metrics, the *k*-mer completeness and QV consensus quality values were calculated in Merqury ( Rhie *et al.*, 2020). This work was done using Nextflow ( Di Tommaso *et al.*, 2017) DSL2 pipelines “sanger-tol/readmapping” ( Surana *et al.*, 2023a) and “sanger-tol/genomenote” ( Surana *et al.*, 2023b). The genome was analysed within the BlobToolKit environment ( Challis *et al.*, 2020) and BUSCO scores ( Manni *et al.*, 2021; Simão *et al.*, 2015) were calculated.

Table 3 contains a list of relevant software tool versions and sources.

**Table 3.** Software tools: versions and sources.

Software tool	Version	Source
BlobToolKit	4.2.1	https://github.com/blobtoolkit/blobtoolkit
BUSCO	5.3.2	https://gitlab.com/ezlab/busco
Hifiasm	0.16.1	https://github.com/chhylp123/hifiasm
HiGlass	1.11.6	https://github.com/higlass/higlass
Merqury	MerquryFK	https://github.com/thegenemyers/MERQURY.FK
MitoHiFi	3.01	https://github.com/marcelauliano/MitoHiFi
OATK		https://github.com/c-zhou/oatk
PretextView	0.2	https://github.com/wtsi-hpag/PretextView
sanger-tol/genomenote	v1.0	https://github.com/sanger-tol/genomenote
sanger-tol/readmapping	1.1.0	https://github.com/sanger-tol/readmapping/tree/1.1.0
YaHS	1.1a.2	https://github.com/c-zhou/yahs

### Wellcome Sanger Institute – Legal and Governance

The materials that have contributed to this genome note have been supplied by a Darwin Tree of Life Partner. The submission of materials by a Darwin Tree of Life Partner is subject to the **‘Darwin Tree of Life Project Sampling Code of Practice’**, which can be found in full on the Darwin Tree of Life website here. By agreeing with and signing up to the Sampling Code of Practice, the Darwin Tree of Life Partner agrees they will meet the legal and ethical requirements and standards set out within this document in respect of all samples acquired for, and supplied to, the Darwin Tree of Life Project. 

Further, the Wellcome Sanger Institute employs a process whereby due diligence is carried out proportionate to the nature of the materials themselves, and the circumstances under which they have been/are to be collected and provided for use. The purpose of this is to address and mitigate any potential legal and/or ethical implications of receipt and use of the materials as part of the research project, and to ensure that in doing so we align with best practice wherever possible. The overarching areas of consideration are:

•      Ethical review of provenance and sourcing of the material

•      Legality of collection, transfer and use (national and international)

Each transfer of samples is further undertaken according to a Research Collaboration Agreement or Material Transfer Agreement entered into by the Darwin Tree of Life Partner, Genome Research Limited (operating as the Wellcome Sanger Institute), and in some circumstances other Darwin Tree of Life collaborators.

## Data Availability

European Nucleotide Archive: *Ophion costatus*. Accession number PRJEB61491; https://identifiers.org/ena.embl/PRJEB61491 ( Wellcome Sanger Institute, 2023). The genome sequence is released openly for reuse. The *Ophion costatus* genome sequencing initiative is part of the Darwin Tree of Life (DToL) project. All raw sequence data and the assembly have been deposited in INSDC databases. The genome will be annotated using available RNA-Seq data and presented through the Ensembl pipeline at the European Bioinformatics Institute. Raw data and assembly accession identifiers are reported in Table 1.

## Author information

Members of the Natural History Museum Genome Acquisition Lab are listed here: https://doi.org/10.5281/zenodo.7139035.

Members of the Darwin Tree of Life Barcoding collective are listed here: https://doi.org/10.5281/zenodo.4893703.

Members of the Wellcome Sanger Institute Tree of Life Management, Samples and Laboratory team are listed here: https://doi.org/10.5281/zenodo.10066175.

Members of Wellcome Sanger Institute Scientific Operations: Sequencing Operations are listed here: https://doi.org/10.5281/zenodo.10043364.

Members of the Wellcome Sanger Institute Tree of Life Core Informatics team are listed here: https://doi.org/10.5281/zenodo.10066637.

Members of the Tree of Life Core Informatics collective are listed here: https://doi.org/10.5281/zenodo.5013541.

Members of the Darwin Tree of Life Consortium are listed here: https://doi.org/10.5281/zenodo.4783558.
