# Supplementary material for: The genome sequence of an ichneumonid wasp, Ophion costatus Ratzeburg, 1848
Source: Wellcome Open Res. 2024 Apr 12;9:191. [Version 1] doi: 10.12688/wellcomeopenres.21216.1 (PMC11576570; doi:10.12688/wellcomeopenres.21216.1)
Supplement: Peer review history

# Reviewer report: Reviewer response for version 1

**DOI:** 10.21956/wellcomeopenres.23463.r95102

**Published:** 2024-11-19

**Authors:** Annabel Whibley

**Affiliations:** 1 The University of Auckland, Auckland, Auckland, New Zealand; 2 Bragato Research Institue, Lincoln, New Zealand

Broad, Fletcher, Januszczak and colleagues present a high-quality reference genome assembly of the ichneumonid  wasp *Ophion costatus*. Although RNA extraction was performed, a gene annotation has not yet been released. The taxonomic history of this species is complex but is very carefully set out in the introduction, along with an overview of morophology/phenotype, distribution and other pertinent aspects of natural history.

 As is standard for these Data Notes,  the reference is high-quality, and has been constructed using appropriate tools and with comprehensive reporting of the sample collection, data generation and analysis and all associated metadata. Public links to data accessions and interactive plots are working.

 Relative to other assemblies that I have seen processed through this pipeline, the final assembly (as shown in Figure 3) does seem to contain a number of small scaffolds/contigs that have distinct GC/coverage properties from the bulk of the assembly. The BUSCO scores are <95% completeness, which is, in my experience, a little low but might reflect the quality of the Hymenoptera ODB gene set compared to that of Lepidoptera, which is where I tended to review. Similarly the degree of manual curation also suggests challenges with the sample or genome. But, overall these should not detract from the value and utility of this high-quality reference genome.

Are sufficient details of methods and materials provided to allow replication by others?

Yes

Is the rationale for creating the dataset(s) clearly described?

Yes

Are the datasets clearly presented in a useable and accessible format?

Yes

Are the protocols appropriate and is the work technically sound?

Yes

Reviewer Expertise:

Genomics. Bioinformatics

I confirm that I have read this submission and believe that I have an appropriate level of expertise to confirm that it is of an acceptable scientific standard.

# Reviewer report: Reviewer response for version 1

**DOI:** 10.21956/wellcomeopenres.23463.r88603

**Published:** 2024-07-29

**Authors:** Željko Tomanović

**Affiliations:** 1 Serbia University of Belgrade, Belgrade, Serbia

The authors presented a genome assembly of the male *Ophion costatus*, an ichneumon wasp, including the mitochondrial genome. The background to the biology and ecology of *Ophion costatus* is described in detail. Obviously, the species belongs to a taxonomic group with a complicated taxonomic history and several synonymizations. The presented genomes could help to understand the mechanisms of speciation and host shift.

Are sufficient details of methods and materials provided to allow replication by others?

Yes

Is the rationale for creating the dataset(s) clearly described?

Yes

Are the datasets clearly presented in a useable and accessible format?

Yes

Are the protocols appropriate and is the work technically sound?

Yes

Reviewer Expertise:

diversity, taxonomy and ecology insect parasitoids

I confirm that I have read this submission and believe that I have an appropriate level of expertise to confirm that it is of an acceptable scientific standard.
